# Real-World Utilization of Target- and Immunotherapies for Lung Cancer: A Scoping Review of Studies Based on Routinely Collected Electronic Healthcare Data

**DOI:** 10.3390/ijerph18147679

**Published:** 2021-07-19

**Authors:** Andrea Spini, Giulia Hyeraci, Claudia Bartolini, Sandra Donnini, Pietro Rosellini, Rosa Gini, Marina Ziche, Francesco Salvo, Giuseppe Roberto

**Affiliations:** 1 INSERM, BPH, U1219, Team Pharmacoepidemiology, University of Bordeaux, 33000 Bordeaux, France; francesco.salvo@u-bordeaux.fr; 2 Department of Medical Science, Surgery and Neuroscience, University of Siena, 53100 Siena, Italy; marina.ziche@unisi.it; 3 Osservatorio di Epidemiologia, Agenzia Regionale di Sanità Della Toscana, 50141 Florence, Italy; giulia.hyeraci@ars.toscana.it (G.H.); claudia.bartolini@ars.toscana.it (C.B.); rosa.gini@ars.toscana.it (R.G.); giuseppe.roberto@ars.toscana.it (G.R.); 4 Department of Life Sciences, University of Siena, 53100 Siena, Italy; 5 CIC1401, CIC Bordeaux, 33000 Bordeaux, France; rosellini.pietro@u-bordeaux.fr; 6 Pole de Santé Publique, Service de Pharmacologie Médicale, Centre de Pharmacovigilance de Bordeaux, CHU de Bordueax, 33000 Bordeaux, France

**Keywords:** electronic healthcare data, big data, real-word data, real-word evidence, drug utilization, lung cancer, immunotherapy, target-therapy, scoping review

## Abstract

Routinely collected electronic healthcare data (rcEHD) have a tremendous potential for enriching pre-marketing evidence on target- and immunotherapies used to treat lung cancer (LC). A scoping review was performed to provide a structured overview of available rcEHD-based studies on this topic and to support the execution of future research by facilitating access to pertinent literature both for study design and benchmarking. Eligible studies published between 2016 and 2020 in PubMed and ISI Web of Science were searched. Data source and study characteristics, as well as evidence on drug utilization and survival were extracted. Thirty-two studies were included. Twenty-six studies used North American data, while three used European data only. Thirteen studies linked ≥1 data source types among administrative/claims data, cancer registries and medical/health records. Twenty-nine studies retrieved cancer-related information from medical records/cancer registries and 31 studies retrieved information on drug utilization or survival from medical records or administrative/claim data. Most part of studies concerned non-small-cell-LC patients (29 out of 32) while none focused on small-cell-LC. Study cohorts ranged between 85 to 81,983 patients. Only two studies described first-line utilization of immunotherapies. Results from this review will serve as a starting point for the execution of future rcEHD-based studies on innovative LC pharmacotherapies.

## 1. Introduction

Lung cancer is the most commonly diagnosed cancer worldwide (2.09 million cases in 2018). It accounts for 14.5% of the total cases of cancer in men and 8.4% in women, being the leading cause of cancer death in men (22.0%) [1,2].

Based on histological characteristics, the World Health Organization classifies lung cancers in small-cell lung cancers (SCLC) and non-small-cell lung cancers (NSCLC) [3]. In 2018, SCLC accounted for about 300,000 cases while NSCLC for 1.8 million [1,2]. The latter can be distinguished in two main histotypes: squamous and non-squamous carcinoma [2,4,5,6].

Treatment of lung cancer relies on one or more therapeutic approaches among surgery, radiation therapy and pharmacotherapy [7]. Currently, a wide range of medications is available for the treatment of advanced NSCLC. The choice of a specific pharmacological regimen is mainly based on the stage of the cancer, although other factors such as the overall patient’s health and lung function, as well as some specific molecular traits of the cancer itself, are also important. Early-stage NSCLC shows no overt clinical symptoms, and surgery represents the treatment of choice. In such cases, pharmacotherapy can be used both before, as neoadjuvant treatment aimed to reduce the size of the tumor, and after surgery, as adjuvant treatment intended to decrease the risk of cancer recurrence [5,8]. In advanced stages, where cancer has already spread, treatment choice depends on the specific site and number of metastases, other than age and overall health status of the patient. In particular, while pharmacotherapy of SCLC is based mainly on standard chemotherapy (the FDA approved nivolumab in August 2018), during the last 15 years, the pharmacological treatment for advanced stage NSCLC was revolutionized by the authorization of innovative anticancer therapies, such as target therapy and immunotherapy [9].

Target therapy includes drugs that can counteract specific mechanisms underlying the development of tumors [10]. These include the neutralizing antibody bevacizumab, which acts by binding the pro-angiogenic vascular endothelial growth factor (VEGF), the tyrosine kinase inhibitors (TKIs) directed to the epithelial growth factor receptor (EGFR), and TKIs of anaplastic lymphoma (ALK). Immunotherapy includes nivolumab (approved in 2015) and pembrolizumab (approved in 2016): these drugs inhibit the binding between lymphocyte protein death 1 (PD-1) and tumor ligand of PD-1 (PD-L1) by maintaining the immune system’s response to the tumor [11,12]. In advanced NSCLC and in non-operable patients, some of these drugs are the first-line treatment (e.g., anti-TKIs) in patients with activating mutations in EGFR or ALK genes, while others are licensed as second-line treatment (e.g., Nivolumab) [8,13,14,15]. No target therapies are approved for the treatment of advanced SCLC.

Knowledge on efficacy and safety of authorized anticancer drugs mostly relies on evidence from clinical trials [16]. Such studies are usually based on relatively small samples of strictly selected, well monitored, patient populations, which are generally followed for short time periods [17].

In this context, observational studies based on large databases of routinely collected electronic healthcare data (rcEHD) has the potential to complement information from clinical trials by allowing the observation of the “real world” clinical practice, thus leveraging data from wider and less strictly selected populations, during long-term follow-up periods [18,19]. Given also the hot topic of using big data, as well as artificial intelligence, for longitudinal data mining in healthcare [20], an overview of available data to conduct pharmacoepidemiologic studies is needed. Unfortunately, the conduction of such studies in the oncology setting remains often a challenge since information to reliably describe utilization of cancer drugs, patients’ characteristics and outcomes are often scattered in distinct data sources.

We performed a scoping review [21] of the published rcEHD-based studies concerning the utilization of target- and immuno-therapies in LC patients with the aim of providing a structured overview of the available studies to facilitate the design and benchmark of future works on this topic.

## 2. Material and Methods

### 2.1. Literature Search

We searched PubMed and ISI Web of Science databases for retrieving the articles of interest that were published from January 2016 to August 2020. Due to the approval of immunotherapy in 2015, January 2016 was chosen as starting date to give a more up-to-date picture of the issue [11]. The search string used was composed by three sets of keywords respectively related to the concepts “lung cancer”, “drug-utilization measures”, and “type of study/Data”, respectively (see Supplementary Materials—Table S1 for more details). Snowballing search was also conducted to retrieve additional papers of interest by examining the references cited in the included articles.

### 2.2. Eligibility Criteria

Retrospective observational studies based on information retrieved from rcEHD that reported evidence on target therapies and immunotherapies in patients with lung cancer were selected. Eligible studies had to be published between January 2016 and August 2020 and written in English. Studies based on ad hoc data collection or with no abstract or full-text available were excluded.

### 2.3. Study Selection

Two authors (AS and GH) screened all titles and abstracts of the references retrieved. Potentially relevant studies were further assessed through examination of full texts. The reviewers worked independently, in parallel, and blinded to each other. Disagreements between the two reviewers were solved through discussion with a third author (GR).

### 2.4. Data Extraction

The following information was extracted from the included studies:

(i) Data source characteristics: type of source, name, catchment area. Notably, data source types were classified into three main categories: (a) administrative/claims data (i.e., data for health system planning and management and health assistance claims), (b) “medical/health records” (i.e., documentation of clinical care) and (c) “cancer registries” [22,23];
(ii) Study characteristics: study population, population size, cohort type (population-based, hospital-based), study period, follow-up duration and drugs or drugs regimens. Additionally, relevant information items such, as cancer-related characteristics, patients-related characteristics, drug utilizations, vital status, were also classified by sources of rcEHD used, whenever possible;
(iii) Information on the utilization of target- and immunotherapies based on treatment line and LC histology (e.g., pattern of use, frequency molecular testing, survival).

As for study selection, two authors extracted independently the data (AS and GH), and a third author (GR) was consulted in case of disagreement.

In particular, median overall survival (OS) values were extracted, whenever reported. Median OS values were grouped by treatment line and presented as the range between the maximum and minimum reported value.

## 3. Results

### 3.1. Literature Search Results

**Figure 1:**
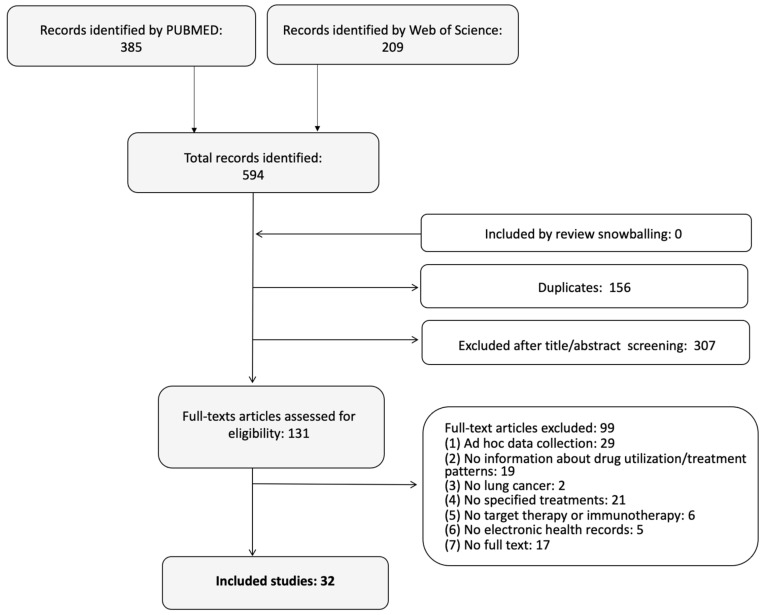
Flow chart.

A total of 594 study references were retrieved from PubMed and ISI Web of Science (Figure 1).

Screening of titles and abstracts allowed the selection of 131 potentially eligible studies. Among them, a total of 32 studies fulfilled the eligibility criteria and were finally included into the review [24,25,26,27,28,29,30,31,32,33,34,35,36,37,38,39,40,41,42,43,44,45,46,47,48,49,50,51,52,53,54,55]. No further studies were retrieved through a snowballing search.

### 3.2. Source of Routinely Collected Electronic Healthcare Data (rcEHD) Used by Study

Twenty-six out of 32 included studies used rcEHD from North America [27,28,29,30,31,33,34,35,36,37,38,39,40,41,43,44,45,46,47,48,49,50,51,52,53,54], two studies used data from Asia [24,32], three from Europe [26,42,55], and one from Australia [25]. Thirteen studies used record linkage of ≥1 type of data source [24,25,26,28,30,32,35,36,42,44,45,50,55], while 19 studies were based on one data source type only. Among the latter, 14 studies used medical/health records only [29,33,34,37,41,43,46,47,48,49,51,52,53,54], four were based on administrative/claim data [31,38,39,40], and one study used data from cancer registries (Table 1) [27]. Four studies [30,44,45,50] linked administrative/claim data with cancer registries.

### 3.3. Characteristics of the Included Studies

The majority of the included studies concerned NSCLC patients (28 out of 32) [24,25,26,27,28,29,30,32,33,34,36,37,38,40,41,42,43,44,45,46,47,48,49,50,51,52,53,54], three studies included patients with unspecified lung tumor [31,39,55], and one study concerned neuroendocrine lung tumor [35]. Patients with SCLC were identified and included in one study, although the latter study primarily concerned NCSLC treatment (Table 2) [27].

The size of the study populations ranged from 85 to 81,983 patients. Sixteen studies included only patients ≥18 years of age [24,28,29,31,33,34,35,38,41,42,43,46,47,51,53,54], five concerned elderly patients only (≥65 years old) [30,32,44,45,50], while 11 studies did not apply any age restriction to the study population [25,26,27,36,37,39,40,48,49,52,55]. Most part of the studies were population-based (28 out of 32) [28,29,30,31,32,33,34,35,36,37,38,39,40,41,42,43,44,45,46,47,48,49,50,51,52,53,54,55], while four were hospital-based [24,25,26,27]. All the included studies were longitudinal. Studies’ observation period ranged between 2000 and 2018 [36,48]. Among studies that reported follow-up duration (13 out of 32), the mean follow-up time ranged from 6.9 to 20 months [44,53].

### 3.4. Sources of rcEHD Used for Information Retrieval

Medical/health records and cancer registries were most frequently used to retrieve cancer-related information (i.e., histology, stage, molecular/genetic characterization, tumor response and disease progression—see Table S2 Supplementary Materials): on a total of 23 studies where the source used to retrieve the reported cancer-related information could be assessed, 13 used medical/health records [25,29,33,37,41,43,47,48,49,51,52,53,54] and seven used cancer registry data [26,27,28,32,36,42,50]. Notably, tumor response was reported in four studies only: the information was always retrieved from medical/health records [25,33,43,48]. In three studies based on administrative/claim data, instead, proxies of cancer-related information were used to identify tumor histology and/or stage [31,39,55]. A study based on French administrative healthcare data used bevacizumab or pemetrexed dispensing as a proxy for non-squamous NSCLC histology [55]. Two other studies based on administrative/claims data from US, identified patients with metastatic cancer by using algorithms based on a combination of ICD-9CM codes (e.g., excluding patients with a claim for lung surgery, and then selected only those patients with ICD-9CM codes referring to a metastatic disease—see Table S3 Supplementary Materials further details on algorithms used to derive missing variables from administrative/claim data) [31,39].

Out of the 32 studies reporting information on drug utilization, 18 studies used medical/health records [25,26,28,29,33,36,37,41,42,43,47,48,49,51,52,53,54] and seven administrative/claim data [30,31,32,38,39,40,55]. Notably, four studies derived information on treatment line from administrative/claims data [31,32,35,55] and 16 from medical/health records [25,26,29,33,37,41,42,46,47,48,49,51,52,53,54].

As for information on vital status, it was retrieved from administrative data in seven studies [28,32,33,34,36,39,55] and from medical/health records in 11 studies [25,37,41,43,47,48,49,51,52,53,54], in a total of 20 studies in which the source used to retrieve vital status could be assessed.

### 3.5. Utilization of Target- or Immuno- Therapies for Non-Small-Cell Lung Cancers (NSCLC)

Twenty-nine out of 32 studies described the use of innovative treatments in patients with NSCLC [24,25,26,28,29,30,32,33,34,37,40,41,42,43,44,45,46,47,48,49,50,51,52,53,54,55].

#### 3.5.1. First-Line Treatments for Advanced NSCLC Patients (III–IV Stage)

Twenty-one studies reporting information on the use of innovative pharmacotherapy as first-line treatment for advanced NSCLC were found [24,25,26,28,29,30,32,33,34,41,42,43,45,46,48,49,50,51,52,53,54]: two studies concerned immunotherapy [52,54], 13 studies concerned the anti-angiogenic drug bevacizumab [24,28,29,30,33,34,41,42,43,45,50,51,54], and fourteen concerned TKIs.

The use of first-line immunotherapy was described by two studies [52,54]. Information about pembrolizumab and nivolumab (e.g., changes in treatment line during study period and trend for utilization) was reported in both studies. The study of Molife et al., reported also that no patients received atezolizumab as a first-line treatment in a population extracted from the US Flatiron healthcare database from 2014 to 2017 [54].

**Figure 2:**
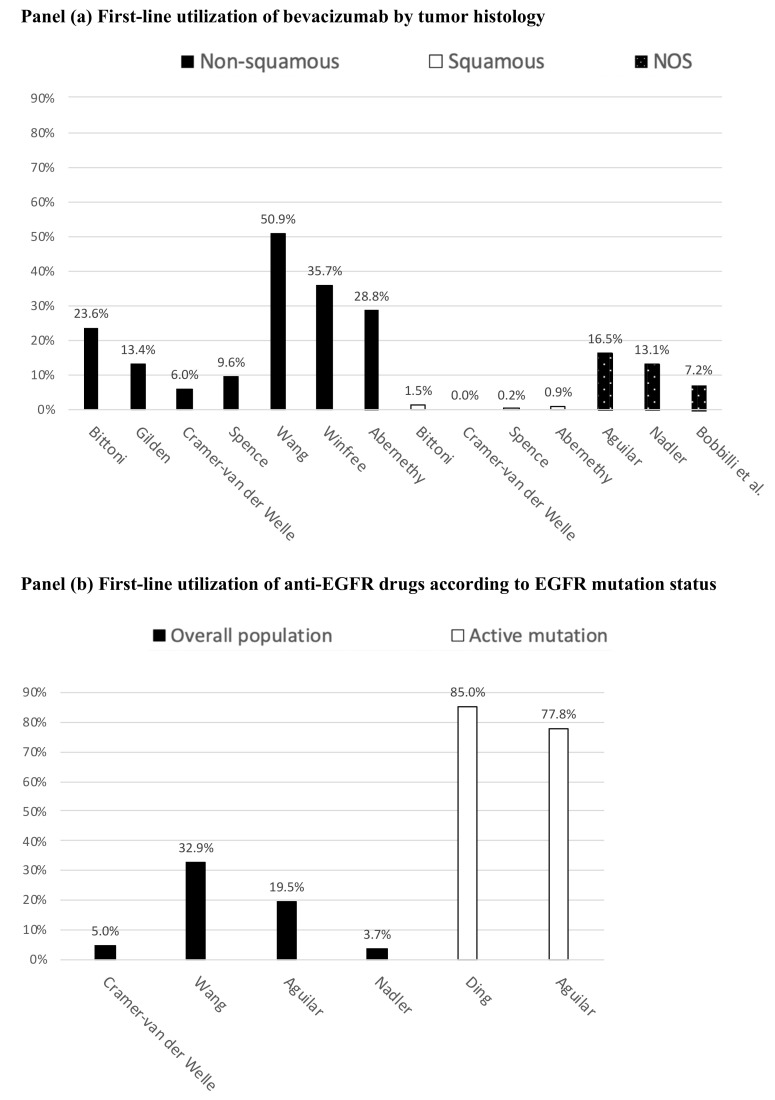
Reported incidence of use of innovative drugs as first-line pharmacotherapy for advanced non-small-cell lung cancers (NSCLC). (**a**) First-line utilization of bevacizumab by tumor histology; (**b**) First-line utilization of anti-EGFR drugs according to EGFR mutation status; NOS: not otherwise specified.

Eight studies described the use of bevacizumab in relation with histology (i.e., squamous/non-squamous) [24,28,29,30,33,42,43,45], while five did not specify the histology of NSCLC [34,41,50,51,54]. The prevalence of use of first-line bevacizumab among patients with advanced NSCLC was reported in 10 studies [24,28,29,30,34,41,42,43,45,50]. Among the latter studies, the use of bevacizumab in non-squamous NSCLC patients, varied between 6.0% and 50.9%, while it was negligible in patients with squamous NSCLC (from 0.0% to 1.5%; see Figure 2a). The study of Molife et al., included also a cohort of patients treated with ramucirumab [54].

Among the 11 studies concerning first-line anti-EGFR TKIs, eight reported the use of erlotinib [24,25,34,41,42,49,50]. Among these, three studies also provided information on the use of gefitinib [24,42,49], and two on the use of afatinib [24,49]. Five studies reported that the incidence of use of anti-EGFR among advanced NSCLC patients varied between 3.7% and 32.9% [24,34,41,42,50]. Two studies described the use of anti-EGFR TKIs in a population of NSCLC patients with an activating mutation of the related gene, and found an incidence of use between 77.8% and 85.0% (Figure 2b) [25,41]. The anti-EGFR TKIs median duration of first-line treatment in patients with activating mutation ranged between 6.5 months and 9 months [25,41]. Four studies concerned the use of the anti-ALK TKI crizotinib [34,46,48,54], three ceritinib [46,48,54], two alectinib [48,54] and one brigatinib [48]. Notably, two out of four studies [46,48] investigated the use of first-line anti-ALK TKIs in a population with ALK-mutated NSCLC.

#### 3.5.2. Second-Line Treatments for Advanced NSCLC (III–IV Stage)

**Figure 3:**
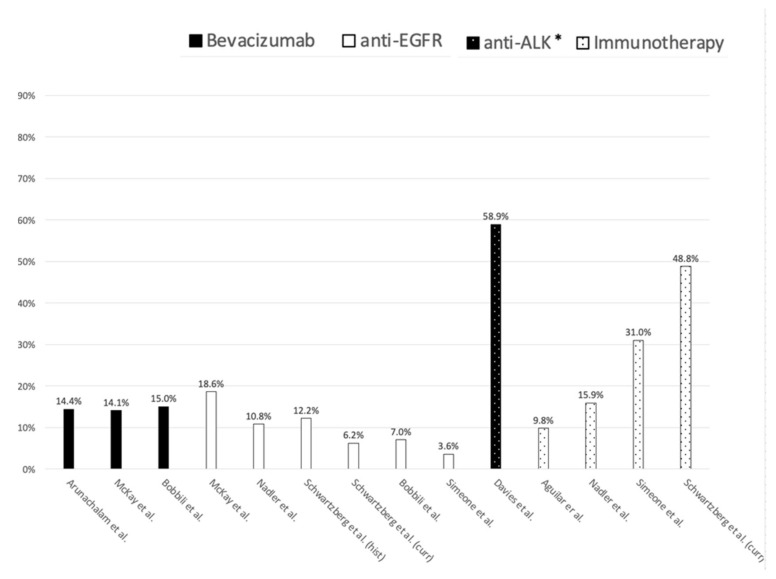
Reported incidence of use of innovative drugs as second-line pharmacotherapy for NSCLC. * Utilization was assessed in patient with anaplastic lymphoma (ALK)-mutated NSCLC.

Thirteen studies described the use of innovative anticancer drugs as second-line pharmacotherapy for advanced NSCLC (Figure 3) [29,33,34,37,41,44,46,48,50,51,52,53,54].

Seven studies described the use of immunotherapy as second-line treatment in patients with NSCLC [29,34,41,51,52,53,54]. PD-L1 cancer expression ranged between 1.3% and 57.7% and was reported in five studies [34,51,52,53,54]. Studies that reported information about the use of nivolumab [34,41,51,52,53,54], pembrolizumab [52,53,54] and atezolizumab [52,53,54] were six, three and three, respectively. One study described immunotherapies utilization without distinction on the active substance concerned [29].

Five studies described the use of bevacizumab as second-line treatment for advanced NSCLC in the US [37,44,50,51,54]. The reported incidence of use varied between 6.2% and 15%. Two studies shows that second-line bevacizumab was used to treat non-squamous NSCLC patients only [37,44].

Eight studies from the US concerned anti-EGFR therapies as second-line in patients with advanced NSCLC [34,37,49,50,51,52,53,54]. All the eight studies described the use of erlotinib, of which three described also the use of gefinitib, afatinib and osimertinib [49,53,54]. Five studies showed that the incidence of use of second-line anti-EGFR utilization among advanced NSCLC patients ranged between 3.6% and 18.6% [34,37,50,51,53]. Four US studies reported the use of anti-ALK therapies as second-line therapy [46,48,52,54]. Two out of four studies described the use of anti-ALK medications in an ALK mutated NSCLC cohort [46,48], while the remaining two studies concerned a cohort of patients included regardless of molecular characteristics of the tumor.

### 3.6. Utilization of Target- or Immuno-Therapies for Neuroendocrine Lung Cancer

One out of the 32 included studies referred to patients with neuroendocrine lung cancer [35]. Using the MarketScan Database and PharMetrics Database between July 2009 and June 2014, the authors reported that in a total of 785 patients, 78.2% started first-line therapy with cytotoxic chemotherapy, 18.1% with somatostatin analogues, and 1.1% with other drugs such as sunitinib or everolimus.

### 3.7. Utilization of Target- or Immuno-Therapies for Unspecified Lung Cancer Histology

Using administrative data only, two studies (two from the US) included advanced stage lung cancer patients regardless whether they were diagnosed with NSCLC or SCLC [31,39,55]. Both studies used data sources from the USA [31,39]: the first described the first-line use of biologic therapy (bevacizumab, crizotinib, erlotinib and cetuximab) in patients with metastatic lung cancer by site-of-care [31] and the second one the use of erlotinib in patients with EGFR mutated metastatic lung cancer [39].

### 3.8. Survival of Patients Treated with Target- or Immuno- Therapies for Advanced NSCLC

Twenty-two out of 32 studies reported the median overall survival (OS) of patients with advanced NSCLC [25,26,27,28,29,30,32,33,34,36,42,43,44,45,46,47,48,50,51,52,53,54]. The shortest median OS was reported for patients with stage IV NSCLC in the period 2011–2013 without known EGFR or ALK mutations (7.3 months), while the longest median OS was reported in patients with ALK activating mutations in a US population during the period 2011–2017 (27.6 months) [47,53]. In patients without EGFR or ALK mutations, the reported median OS ranged from 7.8 to 10 months for non-squamous NSCLC and from 6.5 to 8.5 months for patients with squamous NSCLC [29,53]. Three studies reported evidence on median OS in elderly patients (≥65 years old) [44,45,50] which ranged between 6.4 and 6.7 months for squamous advanced NSCLC, and between 7.5 and 7.8 for non-squamous advanced NSCLC.

Eight studies reported evidence on OS by drug treatment (see Table S4 Supplementary Materials for the range of reported median OS found by treatment among advanced/metastatic NSCLC patients) [28,33,34,42,43,48,51,54]. Among the latter, five were referred to an advanced stage (III–IV) NSCLC [28,33,43,48,54], and three concerned metastatic stage (IV) NSCLC only [34,42,51]. Seven studies reported median OS for first-line drug treatments [28,33,34,42,43,48,54], and three studies also reported median OS for second- or third-line pharmacotherapies [34,48,51,52].

Median progression-free survival (PFS) was reported by five studies [25,33,43,48,52]. Among them, three studies reported PFS in relation to first-line treatment [33,43,48] and two studies in relation to second-line treatments [48,52] (see Table S4 Supplementary Materials).

## 4. Discussion

With this scoping review we provided a structured overview of the available literature concerning recently published rcEHD-based studies concerning the utilization of target- or immunotherapies for LC. Our results highlighted a paucity of studies performed in Europe concerning immunotherapies, particularly as first-line pharmacotherapy, and the absence of papers reporting on the utilization of innovative drugs in SCLC patients. Focusing on the different types of rcEHD and methodologies used to retrieve information, results from this review represent a starting point for future studies on this topic, also highlighting current gaps of knowledge and facilitating access to pertinent literature both for study design and for benchmarking of results.

As for countries of data provenance, most of the studies included in this scoping review were conducted using data from the USA or Canada. This is probably because in regions other than North America, healthcare data are often scattered in different and heterogeneous databases, so that the performing studies on rare events that requires information from different healthcare settings, as in the case of lung cancer, remains a challenge [56]. Moreover, the approval of new anticancer medication in Europe is often delayed compared to the US [57], possibly contributing to the higher number of studies from the US included in this review compared to those using European Union (EU) data.

Indeed, results from this review demonstrated that a unique source of electronic healthcare data among administrative/claims data, medical/health records, cancer registry is often insufficient for performing an observational study on the real-world utilization of drugs for LC, as well as for other types of tumor [58].

Concerning the specific sources of rcEHD used for information retrieval, medical/health records were the most frequently used source of information for assessing drug exposure. Among the studies included in this review, administrative/claims data were less frequently used for retrieving such information. This was probably because in-hospital drug utilization might not always be tracked at patient-level in this type of rcEHD [30,31,32,33,34,36,39,55]. Also information on treatment-line is not usually available in administrative/claims data, although ad hoc algorithms can be adopted to derive this information (Table S3 Supplementary Materials) [31,32,35,55]. Moreover, administrative/claims data usually do not record clinical information, such as tumor stage, histology, or gene mutations, which are crucial for studying drug utilization patterns and health outcomes in cancer patients. In this respect, the use of medical/health records or cancer registries appeared to be in most cases necessary [25,28,29,32]. Our results showed that information on disease progression and tumor response was only retrieved from medical/health records [25], while vital status was assessed using administrative/claims data or medical health records, although the former are usually considered as the gold standard for such information [59]. Indeed, each type of data source has its strengths and limitations with respect to the specific research question that needs to be addressed. Even within each of the three general categories of data sources adopted in this review [19], a significant heterogeneity in terms of content and validity has to be expected (see Table S2 Supplementary Materials). Therefore, as has already happened in other contexts [56,60,61], fostering the development of methodologies for leveraging data diversity will be of paramount importance for the generation of solid evidence on the real-world utilization of drugs in LC.

As for evidence on the real-world utilization of innovative anticancer drugs, most of the included studies concerned patients with advanced stage NSCLC while no studies focusing on SCLC were found. The absence of licensed target therapies and the recent approval of immunotherapies for SCLC (Nivolumab was the first approved in August 2018 in the US [62]) apparently explains the absence of any published study focusing on SCLC in our literature review. Given the very low prevalence of SCLC [8], rcEHD has the potential to play an important role in capturing and studying far larger populations of SCLC patients than those recruited in clinical trials. The orphan designation of different drugs intended for the treatment of SCLC has promoted the study of a number of promising treatments [63], mainly immunotherapies, that were recently marketed, or will be possibly approved in the near future [62,64]. However, further initiatives are desirable to foster SCLC genotyping for the discovery of new molecular targets useful to develop innovative medications. Findings from this review showed that available evidence on immunotherapies from rcEHD-based studies concerning immunotherapy used for advanced stage NSCLC is still scarce, particularly with respect to their use as first-line pharmacotherapy. Notably, only two studies reported evidence on the real-world utilization of immunotherapies administered as first-line pharmacotherapy in patients with advanced NSCLC [52,54]. Such paucity of literature is mostly due to the recent approval of this class of anticancer medications for such indications. In fact, pembrolizumab was the first immunotherapy approved for first-line treatment of advanced NSCLC in 2017 [11,12]. The reported estimates of the incidence of use of immunotherapies as second-line pharmacotherapy for NSCLC, instead, appeared extremely variable from one study to the other mostly due to the different study period, cohort characteristics, and active principles concerned (from 9.8% to 48.8%) [29,34,41,53]. Such heterogeneity of study characteristics and results, however, represents an important resource for benchmarking results of future studies. Conversely, from immunotherapy a markedly higher number of studies on target therapies as first or second-line treatment for NSCLC were found. These studies provided information on the real-world utilization of such a class of medications, such as estimates of the frequency of the use in the relevant study populations, by histology as well as by molecular test execution.

The main strength of this review is the systematic approach adopted for reviewing the available body of recently published literature on the topic, with an in-depth screening of the records retrieved from two comprehensive databases like PubMed and ISI web of science. In particular, the choice of including studies published starting from 2016 was mainly due to the recent approval of some of the drugs and indications of interest (e.g., the first included studies concerning immunotherapies was published in 2017). Moreover, this approach, other than increasing the efficiency of the literature search efforts (i.e., the number of observational studies published increased in the last few years [65]), allowed us to provide an overview of studies concerning the most up-to-date evidence and methodologies on the topic. Indeed, given the scoping nature of this review, quality assessment of the included studies was not performed.

## 5. Conclusions

In conclusion, this scoping review provided a structured overview of the published rcEHD-based studies that investigated the real-world utilization of target and immunotherapies in lung cancer patients. The characteristics of studies included in this review showed that record-linkage of different sources of rcEHD often appears to be necessary. Cancer-related information were mainly retrieved from medical/health records or cancer registries while information on drug utilization or vital status were extracted in most of cases from medical/health records or administrative/claim data. As for evidence collected on the utilization of innovative medications for lung cancer, our results highlighted a paucity of studies performed in Europe as well as concerning immunotherapies, particularly as first-line pharmacotherapy. Notably, no study reporting drug utilization evidence concerning SCLC patients was found due to the absence of licensed target therapies and the very recent approval of immunotherapies for this indication.

Finally, this work will serve as a starting point for the execution of future real-world studies based on rcEHD facilitating access to pertinent literature both for study design and for benchmarking of results.

## Figures and Tables

**Table 1:** Data source characteristics.

Study Reference Study	Data Source Name	Catchment Area	Datasource Type		
			Administrative/Claims Data	Medical/Health Records	Cancer Registry
Dawe et al., 2016 [36]	Institute for Clinical Evaluative Sciences databases:Ontario Cancer Registry, OHIP billing claims data, Ontario Drug Benefit, Hospital Discharge Abstracts, National Ambulatory Care Reporting System, Home Care Database, Ontario vital statistics	Canada	☑	☑	☑
Spence et al., 2017 [28]	Kaiser Permanente California (KPSC)	USA	☑	☑	☑
Cramer-van der Welle et al., 2018 [42]	− Care for Outcome registry (built on the Dutch cancer registry) − Clinical data from Netherland hospitals − Santeon Farmadatabase	Netherlands		☑	☑
Peters et al., 2017 [26]	− Care for Outcome registry (built on the Dutch cancer registry) − Clinical data from Netherland hospitals − Santeon Farmadatabase	Netherlands		☑	☑
Broder et al., 2018 [35]	− Truven Health Analytics MarketScan Database − IMS PharMetrics Database	USA	☑	☑	
Arunachalam et al., 2018 [44]	− Surveillance, Epidemiology and End Results-Medicare database (SEER) − Medicare files	USA	☑		☑
Bittoni et al., 2018 [45]	− Surveillance, Epidemiology and EndResults-Medicare database (SEER) − Medicare files	USA	☑		☑
Bobbili et al., 2019 [50]	− Surveillance, Epidemiology and End Results-Medicare database (SEER) − Medicare files	USA	☑		☑
Gilden et al., 2017 [30]	− Surveillance, Epidemiology and End Results-Medicare database (SEER) − Medicare files	USA	☑		☑
Liang et al., 2016 [32]	Taiwan Cancer Ragistry, National Health Insurance and National Death Registry	Taiwan	☑		☑
Abernethy et al., 2017 [29]	Flatiron Health database	USA		☑	
Aguilar et al., 2018 [41]	US Oncology Network’s iKnowMed database	USA		☑	
Chiang et al., 2020 [49]	Flatiron Health database	USA		☑	
Davies et al., 2019 [46]	Flatiron Health database	USA		☑	
Ding et al., 2017 [25]	Electronic medical records from South Western Sydney Local Health District (SWSLHD)	Australia		☑	
Jahanzeb et al., 2020 [48]	Flatiron Health database	USA		☑	
Khozin et al., 2019 [52]	Flatiron Health database	USA		☑	
Lunacsek et al., 2016 [33]	− International Oncology Network (ION) electronic medical record (EMR) database − Social Security Death Master File	USA		☑	
McKay et al., 2016 [37]	Flatiron Health database	USA		☑	
Molife et al., 2019 [54]	Flatiron Health database	USA		☑	
Nadler et al., 2018 [34]	US oncology iKnowMedTM (iKM) databaseSocial Security Death Index	USA		☑	
Schwartzberg et al., 2019 [53]	Flatiron Health database	USA		☑	
Simeone et al., 2019 [51]	Flatiron Health database	USA		☑	
Waterhouse et al., 2020 [47]	US Oncology Network’s iKnowMed database	USA		☑	
Winfree et al., 2018 [43]	Flatiron Health database	USA		☑	
Kasymjanova et al., 2017 [27]	Jewish General Hospital’s Pulmonary Division Lung Cancer Registry	Canada			☑
Dalal et al., 2018 [38]	− Medical and pharmacy claims of insured employees and their dependents − Medicare-eligible retirees with employer-provided Medicare Supplemental plans	USA	☑		
Hopson et al., 2018 [31]	Humana Research Database	USA	☑		
Levra et al., 2020 [55]	Programme de Médicalisation des Systèmes d’Information (PMSI)	France	☑		
Shen et al., 2017 [39]	Truven Health MarketScan database	USA	☑		
Shinde et al., 2016 [40]	Truven Health MarketScan database	USA	☑		
Wang et al., 2017 [24]	Medical Data Vision Database	Japan	☑		

**Table 2:** Study characteristics.

Study Reference	Study Population, Sample Size Cohort Type	Observation Period	Follow-Up Duration	Drugs or Drug Regimens under Study		
				Target Therapy	Immunotherapy	Other
Dawe et al. [36]	NSCLC 81,983 patients Population-based	January 2000–December 2010	-	Target therapy NOS	-	Standard chemotherapy, Complex single or multi agents, Special single agents or multi agents
Spence et al. [28]	Patients ≥18 years old, NSCLC (III-IV) 2081 patients Population-based	January 2008–September 2014	-	Bevacizumab Erlotinib	-	Carboplatin, Cisplatin, Docetaxel, Etoposide, Gemcitabine, Paclitaxel, Pemetrexed, Vinorelbine
Cramer-van der Welle et al., [42]	Patients ≥18 years old, NSCLC (IV) 1214 patients Population-based	January 2008–December 2014	-	Bevacizumab Erlotinib Gefinitib	-	Carboplatin, Cisplatin, Docetaxel, Gemcitabine, Paclitaxel
Peters et al. [26]	NSCLC (III-IV) 2158 patients Hospital-based	January 2008–December 2012	-	TKI	-	Carboplatin, Cisplatin, Gemcitabine, Pemetrexed
Broder et al. [35]	Patients ≥18 years old; lung neuroendocrine tumor 785 patients Population-based	July 2009–June 2014	14.3 months (Median: 11 months)	Target therapy NOS	-	Cytotoxic chemotherapy, Somatostatin analogues (+/−interferon)
Arunachalam et al. [44]	Patients ≥65 years old, NSCLC (III-IV) 4033 patients Population-based	January 2007–December 2011	20 months; (Median 15.7 months)	Bevacizumab	-	Carboplatin, Cisplatin, Docetaxel, Gemcitabine, Pemetrexed, Vinorelbine
Bittoni et al. [45]	Patients ≥65 years old, NSCLC (III-IV) 5931 patients Population-based	January 2007–December 2011	13.6 months; (Median 8.9 months)	Bevacizumab	-	Carboplatin, Cisplatin, Docetaxel, Gemcitabine, Pemetrexed, Vinorelbine
Gilden et al. [30]	Patients ≥65 years old, NSCLC (IIIB-IV) 77,756 patients Population-based	January 2008–December 2010	-	Bevacizumab	-	Carboplatin, Cisplatin, Paclitaxel, Pemetrexed
Liang et al. [32]	Patients ≥65 years old, NSCLC (advanced) 25,008 patients Population-based	January 2005–December 2009	Median 14 months	Bevacizumab Erlotinib Gefitinib	-	Carboplatin, Cisplatin, Docetaxel, Etoposide, Epirubicin, Gemcitabine, Paclitaxel Pemetrexed, Tegafur, Vinorelbine
Abernethy et al. [29]	Patients ≥18 years old, NSCLC (IV) 4441 patients Population-based	November 2012–January 2015	-	Bevacizumab Erlotinib	PD-1 inhibitors	Carboplatin, Cisplatin, Docetaxel, Etoposide, Gemcitabine, Paclitaxel, Pemetrexed, Vinorelbine
Aguilar et al. [41]	Patients ≥18 years old, NSCLC (metastatic) 3108 patients Population-based	January 2011–June 2015	10.3 months (Median 7.6 months)	Bevacizumab Erlotinib	Nivolumab	Carboplatin, Docetaxel, Pemetrexed
Davies et al. [46]	Patients ≥18 years old NSCLC (IIIB-IV, ALK mutated) 300 patients Population-based	January 2011–December 2014	Median 16.6 months	Ceritinib Crizotinib	-	Carboplatin, Cisplatin, Docetaxel, Gemcitabine, Pemetrexed, Vinorelbine
Ding et al. [25]	NSCLC (advanced, EGFR- mutated) 85 patients Hospital-based	January 2010–June 2016	Median 10.7 months	Afatinib Erlotinib Gefinitib Rociletinib	-	-
Lunacsek et al. [33]	Patients ≥18 years old, NSCLC (advanced/metastatic, non-squamous) 431 patients Population-based	April 2006–July 2013	-	Bevacizumab Cetuximab Erlotinib	-	Carboplatin, Cisplatin, Docetaxel, Gemcitabine, Metotrexate, Paclitaxel, Pemetrexed, Temozolomide, Vinflunine, Vinorelbine
McKay et al. [37]	NSCLC (advanced) 6867 patients Population-based	January 2011–April 2015	-	Bevacizumab Erlotinib	-	Carboplatin, Cisplatin, Docetaxel, Gemcitabine, Paclitaxel, Pemetrexed Vinorelbine
Nadler et al. [34]	Patients ≥18 years old, NSCLC (IV) 10,689 patients Population-based	January 2012–April 2016	Median 6.9 months	Bevacizumab Crizotinib Erlotinib	Nivolumab	Carboplatin, Docetaxel, Gemcitabine, Paclitaxel, Pemetrexed
Winfree et al. [43]	Patients ≥18 years old, NSCLC (advanced, non-squamous) 715 patients Population-based	January 2011–October 2015	Median 13.8 months	Bevacizumab Erlotinib	Nivolumab	Carboplatin, Cisplatin, Gemcitabine, Pemetrexed
Kasymjanova et al. [27]	NSCLC and SCLC 751 patients Hospital-based	January 2010–December 2014	-	Target therapy NOS	-	Chemotherapy: Combined agents, Double agents, Single agent
Dalal et al. [38]	Patients ≥18 years old NSCLC (with at least one prescription for ceritinib) 164 patients Population-based	January 2006–December 2015	-	Ceritinib Crizotinib Other (NOS)	-	Standard chemotherapy
Hopson et al. [31]	Patients 18–89 years old, lung cancer (metastatic) + other tumors 3199 lung cancer patients Population-based	January 2007–December 2013	-	Target therapy NOS	-	Carboplatin, Cisplatin, Docetaxel, Oxaliplatin, Paclitaxel
Shen et al. [39]	lung cancer (advanced) stage 5842 patients Population-based	January 2013–June 2014	-	Bevacizumab Erlotinib	-	Pemetrexed
Shinde et al. [40]	NSCLC (metastatic) 4926 patients Population-based	January 2009–September 2012	-	Crizotinib Erlotinib	-	-
Wang et al. [24]	Patients ≥18 years old, NSCLC (IIIB-IV) 16,413 patients Hospital-based	April 2008–September 2015	10.3 months	Bevacizumab Erlotinib Gefinitib	-	Carboplatin, Cisplatin, Docetaxel, Gemcitabine, Paclitaxel, Tegafur
Waterhouse et al. [47]	Patients ≥18 years old NSCLC (ALK-mutated, with at least one prescription of anti-ALK) 410 patients Population-based	September 2011–December 2017	-	Alectinib Brigatinib Ceritinib Crizotinib	-	-
Jahanzeb et al. [48]	NSCLC (IIIB-IV, ALK-mutated, with at least one prescription of anti-ALK) 581 patients Population-based	January 2011–June 2018	-	Alectinib Brigatinib Ceritinib Crizotinib	-	-
Chiang et al. [49]	NSCLC (metastatic, with at least one prescription of anti-EGFR) 782 patients Population-based	January 2011–September 2017	Median: 10.3 months	Afatinib Erlotinib Gefitinib Osimertinib	Immunotherapy not specified	Chemotherapy not specified
Bobbili et al. [50]	Patients ≥65 years old, NSCLC (III) 4564 patients Population-based	January 2009–December 2014	-	Bevacizumab Erlotinib	-	Carboplatin, Cisplatin, Docetaxel, Etoposide, Gemcitabine, Paclitaxel, Pemetrexed
Simeone et al. [51]	Patients ≥18 years old, NSCLC (IV) 9656 patients Population based	January 2013–January 2017	Median: 8.4 months	Bevacizumab Erlotinib Ramucirumab	Nivolumab	Carboplatin, Cisplatin, Docetaxel, Etoposide, Gemcitabine, Paclitaxel, Pemetrexed, Vinorelbine
Khozin et al. [52]	NSCLC (advanced or progressed, with at least a prescription of immunotherapy) 5257 patients Population based	January 2011–December 2017	-	Alk inhibitors Anti-EGFR Anti-VEGF	Atezolizumab Nivolumab Pembrolizumab	Platinum based chemotherapy, Non platinum based chemotherapy, Single agent Chemotherapy
Schwartzberg et al. [53]	Patients ≥18 NSCLC (advanced or progressed) 6597 patients (2 cohorts): Historical: 2357 patients Current: 4240 patients Population based	Historical: January 2011–December 2013 Current: January 2015–May 2017	Median: 5.8 months	Anti-EGFR Ramucirumab	Atezolizumab Nivolumab Pembrolizumab	Carboplatin, Docetaxel, Gemcitabine, Paclitaxel, Pemetrexed, Vinorelbine
Molife et al. [54]	Patients ≥18 NSCLC (advanced or metastatic, with at least a prescription of immunotherapy or ramucirumab) 4054 Population-based	December 2014–May 2017	-	Afatinib Alectinib Bevacizumab Ceritinib Crizotinib Erlotinib Gefitinib Osimertinib Ramucirumab	Atezolizumab Ipilimumab Nivolumab Pembrolizumab	Carboplatin, Cisplatin, Docetaxel, Gemcitabine, Etoposide, Paclitaxel, Pemetrexed, Vinorelbine
Levra et al. [55]	Patients with NSCLC (with at least a prescription of nivolumab) 10,452 patients Population based	January 2015–December 2016	-	-	Nivolumab Pembrolizumab	Chemotherapy not specified

## Data Availability

The data presented in this study are all available within this manuscript and its Supplementary Materials.

## Supplementary Materials

The following are available online at https://www.mdpi.com/article/10.3390/ijerph18147679/s1, Table S1; Research Strategy, Table S2; Sources of the data used by the included studies per study variable, Table S3; Algorithms used to extract information from administrative/claims data as reported in the papers included in the review, Table S4; Minimum and maximum median OS reported per treatment-line among stage III-IV NSCLC patients.
