# Supplementary material for: Real-World Utilization of Target- and Immunotherapies for Lung Cancer: A Scoping Review of Studies Based on Routinely Collected Electronic Healthcare Data
Source: Int J Environ Res Public Health. 2021 Jul 19;18(14):7679. doi: 10.3390/ijerph18147679 (PMC8305284; doi:10.3390/ijerph18147679)
Supplement: Supplementary file 1 [file ijerph-18-07679-s001.zip › Table S1.pdf]

Table S1. Research strategy

PUBMED

1) ***Lung Cancer****

lung cancer[MeSH Terms] OR lung cancer[Title/Abstract]

2) ***Drug-utilization measure****

pattern of use[Title/Abstract] OR patterns of use[Title/Abstract] OR drug utilization[MeSH Terms] OR drug utilization[Title/Abstract] OR pattern of prescription[Title/Abstract] OR patterns of prescription[Title/Abstract] OR utilization patterns[Title/Abstract] OR utilization pattern[Title/Abstract] OR patterns of utilization[Title/Abstract] OR prescription patterns[Title/Abstract] OR prescription pattern[Title/Abstract] OR prescribing patterns[Title/Abstract] OR prescribing pattern[Title/Abstract] OR persistence[Title/Abstract] OR persistence[Title/Abstract] OR adherence[Title/Abstract] OR discontinuation[Title/Abstract] OR prescribing trends[Title/Abstract] OR prescribing trend[Title/Abstract] OR utilization trends[Title/Abstract] OR utilization trend[Title/Abstract] OR treatment patterns[Title/Abstract] OR utilization trends[Title/Abstract] OR treatment pathway[Title/Abstract] OR treatment pathways[Title/Abstract] OR switch[Title/Abstract]

3) ***Type of study/Data source****

retrospective[Title/Abstract] OR descriptive[Title/Abstract] OR longitudinal OR observational[Title/Abstract] OR electronic health records OR medical records OR medical charts OR electronic database OR record linkage OR administrative data OR administrative databases OR administrative database OR claims database OR cancer registry OR healthcare database OR claim database OR real world[Title/Abstract] OR pharmacy data OR prescription data

4) Filter: English; 2016-2020*

*Blocks were combined using the Boolean operator AND

ISI Web of science

1) Pathology

TS=("lung cancer")

2) Study objective

TS=("pattern of use" OR "patterns of use" OR "drug utilization" OR "pattern of prescription" OR "utilization patterns" OR "utilization pattern" OR "patterns of utilization" OR "prescription pattern" OR "persistence" OR "adherence" OR "discontinuation" OR "prescribing trend" OR "utilization trend" OR "treatment pattern" OR "utilization trends" OR "treatment pathway" OR "treatment pathways" OR "switch")

3) Type of study/Data

TS=("retrospective" OR "descriptive" OR "longitudinal" OR "observational" OR "electronic health records" OR "medical records" OR "medical charts" OR "electronic database" OR "record linkage" OR "administrative data" OR "administrative databases" OR "administrative database" OR "claims database" OR "cancer registry" OR "healthcare database" OR "claim database" OR "real world" OR "pharmacy data" OR "prescription data")

4) Filter: English; Period 2016-2020

*All the four blocks were combined using the Boolean operator AND
