# Supplementary material for: Real-World Utilization of Target- and Immunotherapies for Lung Cancer: A Scoping Review of Studies Based on Routinely Collected Electronic Healthcare Data
Source: Int J Environ Res Public Health. 2021 Jul 19;18(14):7679. doi: 10.3390/ijerph18147679 (PMC8305284; doi:10.3390/ijerph18147679)
Supplement: Supplementary file 1 [file ijerph-18-07679-s001.zip › Table S2.pdf]

Table S2. Sources of the data used by the included studies per study variable.

Study reference	DEMOGRAPHIC CHARACTERISTICS			FOLLOW-UP DURATION	CANCER-RELATED CHARACTERISTICS					PATIENT-RELATED CHARACTERISTICS					PROCEDURES		LUNG CANCER PHARMACOTHERAPY					
	**Age**	**Gender**	**Area/hospital/region**	**Death/end of follow-up**	**Histology**	**Stage**	**Molecular/genetic characterization**	**Tumor response**	**Disease progression**	**Comorbidities**	**Concomitant pharmacotherapies**	**Performance status**	**Smoking status**	**Race**	**Surgery**	**Radiotherapy**	**Prescription**	**Dispensing/administration (Outpatient/Inpatient use)**	**Indication**	**Dose**	**Duration**	**Treatment line**
Spence, 2017	Administrative data	Administrative data	Administrative data	- Administrative data - Cancer registry	Cancer Registry	Cancer registry	–	–	–	ns	–	–	Medical records/Health records	Medical records/Health records	–	–	–	Medical records/Health records	–	–	–	ns
Abernethy, 2017	Medical records/Health records	Medical records/Health records	–	ns	Medical records/Health records	Medical records/Health records	Medical records/Health records	–	–	–	–	–	Medical records/Health records	Medical records/Health records	–	–	Medical records/Health records	–	Medical records/Health records	Medical records/Health records	Medical records/Health records	Medical records/Health records
Wang et al. 2017	ns	ns	ns	ns	ns	ns	ns	–	–	ns	–	–	ns	–	ns	ns	ns	–	–	–	ns	ns
Ding et al. 2017	Medical records/Health records	Medical records/Health records	–	Medical records/Health records	Medical records/Health records	Medical records/Health records	Medical records/Health records	–	Medical records/Health records	–	–	–	Medical records/health records	–	–	–	–	Medical records/Health records	–	–	Medical records/Health records	Medical records/Health records
Gilden et al. 2017	ns	ns	ns	ns	–	ns	–	–	–	ns	ns	–	–	ns	–	–	-	Administrative data	–	-	-	ns
Hopson et al. 2018	Administrative data	Administrative data	Administrative data	–	–	Administrative data	–	–	–	–	–	–	–	Administrative data	–	–	–	Administrative data	-	–	Administrative data	Administrative data
Liang et al. 2016	Cancer registry	Cancer registry	ns	Administrative data	Cancer registry	Cancer registry	–	–	–	–	–	–	–	–	–	–	Administrative data	–	ns	ns	ns	Administrative data
Lunacsek et al. 2016	Medical records/Health records	Medical records/Health records	Medical records/Health records	Administrative data	Medical records/Health records	Medical records/Health records	–	Medical records/Health records	Medical records/Health records	Medical records/Health records	Medical records/Health records	Medical records/Health records	Medical records/Health records	–	Medical records/Health records	Medical records/Health records	Medical records/Health records	–	–	–	Medical records/Health records	Medical records/Health records
Nadler et al. 2018	Medical records/Health records	Medical records/Health records	Medical records/Health records	Administrative data	ns	ns	ns	–	–	–	–	ns	ns	ns	–	–	ns	–	–	–	–	ns
Peters et al. 2017	ns	ns	ns	Cancer registry	Cancer registry	Cancer registry	–	–	ns	Medical records/Health records	–	Medical records/Health records	–	–	–	–	Medical records/Health records	Medical records/Health records	–	Medical records/Health records	Medical records/Health records	Medical records/Health records
Broder et al. 2018	Administrative data	Administrative data	Administrative data	ns	–	–	–	–	–	–	–	–	–	–	–	–	–	Administrative data	–	ns	Administrative data	Administrative data
Dawe et al. 2016	Cancer registry	Cancer registry	–	Administrative data	Cancer registry	ns	–	–	–	Administrative data	–	–	–	–	Administrative data	Administrative data	Medical records/Health records	–	–	–	ns	ns
Kasymjanova et al. 2017	Cancer registry	Cancer registry	Cancer registry	–	Cancer registry	Cancer registry	ns	–	–	–	–	Cancer registry	Cancer registry	–	Cancer registry	Cancer registry	ns	ns	–	–	ns	–
McKay et al. 2016	Medical records/Health records	Medical records/Health records	Medical records/Health records	Medical records/Health records	Medical records/Health records	Medical records/Health records	Medical records/Health records	–	–	–	–	–	Medical records/Health records	Medical records/Health records	–	–	Medical records/Health records	–	–	Medical records/Health records	Medical records/Health records	Medical records/Health records
Dalal et al. 2018	Administrative data	Administrative data	–	ns	–	–	–	–	–	Administrative data	–	–	–	–	Administrative data	Administrative data	Administrative data	–	–	Administrative data	Administrative data	–
Shen 2017 Clin Lung Cancer 2017	Administrative data	Administrative data	Administrative data	Administrative data	–	Administrative data	Administrative data	–	–	Administrative data	–	–	–	–	Administrative data	Administrative data	Administrative data	–	Administrative data	–	–	–
Shinde et al. 2016	Administrative data	Administrative data	–	–	–	–	Administrative data	–	–	–	–	–	–	–	Administrative data	–	Administrative data	–	–	–	–	–
Aguilar et. 2018	Medical records/Health records	Medical records/Health records	Medical records/Health records	Medical records/Health records	Medical records/Health records	Medical records/Health records	Medical records/Health records	–	–	Medical records/Health records	–	Medical records/Health records	Medical records/Health records	Medical records/Health records	–	–	Medical records/Health records	–	Medical records/Health records	Medical records/Health records	Medical records/Health records	Medical records/Health records
Cramer-van der Welle et al. 2018	Cancer registry	Cancer registry	–	Cancer registry	Cancer registry	Cancer registry	Cancer registry	–	–	Medical records/Health records	–	Medical records/Health records	–	–	–	–	Medical records/Health records	Medical records/Health records	–	Medical records/Health records	Medical records/Health records	Medical records/Health records
Winfree et al. 2018	Medical records/Health records	Medical records/Health records	–	Medical records/health records	Medical records/Health records	Medical records/Health records	Medical records/Health records	Medical records/Health records	Medical records/Health records	–	–	Medical records/Health records	Medical records/Health records	Medical records/Health records	–	–	Medical records/Health records	–	Medical records/Health records	Medical records/Health records	Medical records/Health records	Medical records
Arunchalam et al. 2018	Cancer registry	Cancer registry	–	–	ns	ns	ns	–	–	–	–	–	–	ns	ns	–	–	ns	–	–	ns	ns
Bittoni et al. 2018	Cancer registry	Cancer registry	–	–	ns	ns	ns	–	–	–	–	–	–	ns	ns	–	–	ns	–	–	ns	ns
Davies et al.. 2018	Medical records/Health records	Medical records/Health records	–	ns	ns	ns	Medical records/Health records	ns	ns	–	–	ns	Medical records/Health records	ns	–	–	ns	–	ns	Medical records/Health records (physician's notes)	Medical records/Health records (physician's notes)	Medical records/Health records
Waterhouse et al. 2020	Medical records/Health records	Medical records/Health records	–	Medical records/Health records	Medical records/Health records	Medical records/Health records	Medical records/Health records	–	–	–	–	Medical records/Health records	Medical records/Health records	Medical records/Health records	–	–	Medical records/Health records	–	–	–	Medical records/Health records	Medical records/Health records

Jahnzeb et al. 2020	Medical records/Health records	Medical records/Health records	–	Medical records/Health records	Medical records/Health records	Medical records/Health records	Medical records/Health records	Medical records/Health records	Medical records/Health records	–	–	Medical records/Health records	Medical records/Health records	Medical records/Health records	–	–	Medical records/Health records	–	–	–	Medical records/Health records	Medical records/Health records
Chiang et al. 2020	Medical records/Health records	Medical records/Health records	–	Medical records/Health records	Medical records/Health records	Medical records/Health records	Medical records/Health records	–	–	–	–	Medical records/Health records	Medical records/Health records	Medical records/Health records	–	–	Medical records/Health records	–	–	–	–	Medical records/Health records
Bobbili et al. 2019	Cancer registry	Cancer registry	–	–	Cancer registry	Cancer registry	ns	–	–	Administrative data	ns	ns	–	ns	Cancer registry / administrative data	–	–	ns	–	–	Administrative data	–
Simeone et al. 2019	Medical records/Health records	Medical records/Health records	Medical records/Health records	Medical records/Health records	–	Medical records/Health records	Medical records/Health records	–	Medical records/Health records	Medical records/Health records	Medical records/Health records	Medical records/Health records	Medical records/Health records	Medical records/Health records	–	–	Medical records/Health records	–	–	–	Medical records/Health records	Medical records/Health records
Khozin et al. 2019	Medical records/Health records	Medical records/Health records	Medical records/Health records	Medical records/Health records	Medical records/Health records	Medical records/Health records	Medical records/Health records	–	Medical records/Health records	–	–	–	Medical records/Health records	Medical records/Health records	–	–	Medical records/Health records	–	–	–	Medical records/Health records	Medical records/Health records
Schwartzberg et al.	Medical records/Health records	Medical records/Health records	Medical records/Health records	Medical records/Health records	Medical records/Health records	Medical records/Health records	Medical records/Health records	–	Medical records/Health records	–	–	Medical records/Health records	Medical records/Health records	Medical records/Health records	–	–	Medical records/Health records	–	–	–	Medical records/Health records	Medical records/Health records
Molife *et al.*	Medical records/Health records	Medical records/Health records	Medical records/Health records	Medical records/Health records	Medical records/Health records	Medical records/Health records	Medical records/Health records	–	Medical records/Health records	–	–	Medical records/Health records	Medical records/Health records	Medical records/Health records	–	–	Medical records/Health records	–	–	Medical records/Health records	Medical records/Health records	Medical records/Health records
Giaj Levra *et al.*	Administrative data	Administrative data	–	Administrative data	Administrative data	–	–	–	–	Administrative data	–	–	–	–	Administrative data	–	–	Administrative data	–	–	Administrative data	Administrative data

N.S.: not specified, meaning that the variable is used in the relevant study but the source of data is not specified.
