# Supplementary material for: Real-World Utilization of Target- and Immunotherapies for Lung Cancer: A Scoping Review of Studies Based on Routinely Collected Electronic Healthcare Data
Source: Int J Environ Res Public Health. 2021 Jul 19;18(14):7679. doi: 10.3390/ijerph18147679 (PMC8305284; doi:10.3390/ijerph18147679)
Supplement: Supplementary file 1 [file ijerph-18-07679-s001.zip › Table S3.pdf]

Table S3. Algorithms used to extract information from administrative/claims data as reported in the paper included in the review*

Study reference	CANCER-RELATED CHARACTERISTICS			LUNG CANCER PHARMACOTHERAPY				PROCEDURES		Adverse events
	Histology	Molecular characterization	Stage	Diagnosis of lung cancer	Dispensings	Duration	Treatment line	Radiotherapy	Surgery	
Dawe *et al*. [36]	-	-	-	ICD-9CM: 162.2-162.9	Billing codes OHIP - Chemotherapy: G381, G345, G359, G382, G388	-	-	Billing codes OHIP: X310-313	Billing codes OHIP: M142-145	-
Spence *et al*. [28]	-	-	-	-	-	-	-	-	-	The authors provided a full list of ICD-9CM codes used to identify adverse events
Broder *et al*. [35]	-	-	-	Neuroendocrine lung cancer: ICD-9CM 209.21, 209.61	ns	ns	First-line therapy: pharmacologic treatment regimen observed on, or with- in 3 months of the index date (tumor diagnosis).<br>Second-line therapy: treatment was switched from one category of pharmacotherapy to another	-	-	-
Gilden *et al*. [30]	-	-	-	-	ns	-	-	-	-	-
Liang *et al*. [32]	-	-	-	-	ns	-	All lines of chemotherapy were defined according to the inclusion of non-platinum drugs. Drugs prescribed continuously with no more than 2 months interruption were considered same-line therapy.			
Dalal *et al*. [38]	-	-	-	ICD-9CM: 162.2x-162.9x and ICD- 10 CM codes C34.xx	ns	ns	-	-	-	-
Hopson *et al*. [31]	-	-	**Metastatic tumor:** Patients were identified by at least two metastatic ICD-9-CM codes (196.0x–196.1x, 196.3x–196.5x, 196.8x, 197.0x–197.3x, 197.xx, 198.xx) occurring at most 60 days apart any time between 180 days prior to and up to 120 days after their index infusion date	ICD9-CM: 162.2x–162.9x, 163.xx<br>.	.	First-line therapy start date: date of the first eligible drug administration occurring in the study period; first-line therapy end date was defined as the 30 days after a patient's last eligible drug administration occurring prior to a gap in eligible drug therapy of !90 days during the study period	First-line therapy regimens were defined as the class of chemotherapeutic or biologic agents administered on the index infusion date (+7 days)	-	-	-
Shen *et al*. [39]	-	Testing of EGFR: (CPT) code 81235	**Metastatic tumor**: exclusion of patients with a claim for lung cancer surgery. CPT: 32440, 32442, 32445, 32480, 32482, 32484, 32486, 32488, 32500, 32503, 32504, 32520, 32522, 32525, 32657, and 32663<br>Further restriction: patients with ICD-9 codes indicating secondary malignant neoplasms (197.4-198.7, 198.81, 198.82) within 6 months of diagnosis	ICD-9CM: 162.00 - 162.9	We captured the use of erlotinib by NDCs: 50242-0062-01, 50242-0063- 01, 50242-0064-01, 54868-5290-00, 54868-5447-00, and 54868- 5474-00.	-	-	-	-	-
Shinde *et al*. [40]	Considering only patients receiving erlotinib and crizotinib which are only indicated for NSCLC they considered these patients as NSCLC	CPT codes classified as molecular pathology stacking codes (83890-83914) or array codes (88384-88386	-	ICD-9CM: 162.2-162.5, 162.8-162.9	NDC: 54868- 5290, 54868-5447, 54868-5474, 50242-062, 50242-063, or 50242-064 for erlotinib; 0069-8140 or 0069-8141 for crizotinib	-	-	-	-	-
Wang *et al*. [24]	-	-	-	-	--	Time of initiation was the date of the first dose or prescription of first-line, second-line, or third-line therapy. The end of line of therapy (LOT) was defined as the date of the last infusion or, for oral agents, the projected completion date of the prescribed course in the LOT	The first line of therapy (LOT) included the systemic agents initially given during the first 28 days after treatment initiation. The initiation of the first agent had to be within 6 months following the diagnosis of advanced or recurrent NSCLC (index date). The treatment was considered advanced to the second or third LOT, respectively, when: (1) following 4 or more cycles of first or second LOT and a time gap without chemotherapy and/or biologic treatment be- tween 2 consecutive cycles of 46 weeks, a new LOT was created; or (2) there were o4 cycles of first or second LOT and evidence of subsequent administra- tion	-	-	-

						or death, whichever came first. The duration of the first LOT included any subsequent maintenance therapy.	of a new treatment regimen, not including any agent from the first or second LOT, regardless of the time since the end of first or second LOT.			
Bobbili *et al.* [50]	-	-	-	ICD-10CM: C34.0–C34.9<br>ICD-O-3: morphology codes 8012, 8046, 8070, 8071, 8140, 8250, 8480, 8481, 8490 and 8570	-	-	-	-	-	-
Levra *et al.* [55]	The histological type of NSCLC (Sq or NSq) was identified through the proxy measure of a previous specific treatment for NSq-NSCLC, namely bevacizumab or pemetrexed	-	-	ICD-10CM: C34*	-	-	-	-	-	-

CPT: Current procedural terminology; **NDC:** National Drug Codes
