# Supplementary material for: Real-World Utilization of Target- and Immunotherapies for Lung Cancer: A Scoping Review of Studies Based on Routinely Collected Electronic Healthcare Data
Source: Int J Environ Res Public Health. 2021 Jul 19;18(14):7679. doi: 10.3390/ijerph18147679 (PMC8305284; doi:10.3390/ijerph18147679)
Supplement: Supplementary file 1 [file ijerph-18-07679-s001.zip › Table S4.pdf]

Table S4. Minimum and maximum median OS reported per treatment-line among stage III-IV NSCLC patients

First-line treatments	Min-max reported median OS, months
Bevacizumab alone or with chemotherapy (4 studies) [28,33,42,43]	8.2-23.0
Bevacizumab + pemetrexed (2 studies) [28,43]	8.5-23.0
Bevacizumab + carboplatin + paclitaxel (two studies) [33,42]	8.2-11.9
Ramucirumab (one study) [54]	15.1
TKIs (two studies) [28,34]	12.7-24.3
Erlotinib (one study) [42]	14.3
Gefitinib (one study) [42]	21.2
Anti-ALK TKIs (one study) [48]	25.8
Immunotherapy (one study) [54]	23.1
Chemotherapy (five studies) [28,33,34,42,43]	4.9-20.6
**Second-line treatment**	
TKIs, (one study) [34]	11.2
Anti-ALK TKIs (one study) [48]	24.6
Immunotherapy, (one study) [34]	9.7
Nivolumab (squamous NSCLC, one study) [51]	9.2
Nivolumab (non-squamous NSCLC, one study) [51]	12.2
Pembrolizumab (one study) [51]	12
Atezolizumab (one study) [51]	13.8
Chemotherapy, (one study) [34]	9.2
**Third-line treatment**	
TKIs (one study) [34]	7.4
Immunotherapy (one study) [34]	11.3
Chemotherapy (one study) [34]	8.0
